# Efficacy and Evidence Certainty of High‐Frequency rTMS for Cognitive Impairment Across Neuropsychiatric Disorders: An Umbrella Review

**DOI:** 10.1002/brb3.71695

**Published:** 2026-08-17

**Authors:** Lin‐ling Wu, Xin‐cheng Huang, Juan‐juan Bi, Pei‐yuan He

**Affiliations:** ^1^ Unit 2—Department of Female Acute Psychiatry Disorders The Fourth People's Hospital of Chengdu Chengdu Sichuan China; ^2^ Department of Cardiology and Angiology The Fourth People's Hospital of Chengdu Chengdu Sichuan China; ^3^ Department of Health Management Center, Sichuan Provincial People's Hospital, School of Medicine University of Electronic Science and Technology of China Chengdu Sichuan China

**Keywords:** cognitive impairment, evidence grading, meta‐analysis, neuropsychiatric disorders, repetitive transcranial magnetic stimulation, umbrella review

## Abstract

**Background:**

High‐frequency repetitive transcranial magnetic stimulation (HF‐rTMS) is a promising intervention for cognitive impairment across neuropsychiatric disorders. However, existing evidence remains fragmented within disease‐specific reviews, lacking cross‐disorder synthesis and rigorous evaluation of evidence certainty.

**Objective:**

This umbrella review systematically evaluates the efficacy of HF‐rTMS on cognitive function across six neuropsychiatric disorders with sufficient meta‐analytic evidence on cognitive outcomes, including Alzheimer's disease, vascular cognitive impairment, Parkinson's disease, schizophrenia spectrum disorders, major depressive disorder, and attention‐deficit/hyperactivity disorder—and assesses the certainty of evidence.

**Methods:**

We systematically searched CNKI, Cochrane Library, Embase, PubMed, and Web of Science from inception to March 1, 2026, for systematic reviews or meta‐analyses reporting cognitive outcomes. Re‐meta‐analyses were performed using a random‐effects model to calculate standardized mean differences (SMDs) with 95% confidence intervals (CIs). Study quality was appraised via AMSTAR‐2, and evidence certainty was graded using the Grading of Recommendations Assessment, Development and Evaluation (GRADE) framework. The corrected covered area (CCA) was calculated to quantify overlap of primary studies across reviews.

**Results:**

Twenty‐nine meta‐analyses comprising 54 outcome indicators met the inclusion criteria. Pooled analysis indicated that HF‐rTMS significantly improved global cognitive function (SMD = 0.68, 95% CI: 0.53–0.83, p < 0.001), though with substantial heterogeneity (*I* ^2^ = 88.5%). Subgroup analyses highlighted distinct disease‐specific effects: the most robust benefits were observed in Alzheimer's disease (SMD = 0.91) and vascular cognitive impairment (VCI) (SMD = 0.81). We found small‐to‐moderate effects in schizophrenia spectrum disorders (SSD) and Attention‐Deficit/Hyperactivity Disorder (ADHD), but no significant improvements for Parkinson's disease or major depressive disorder. Domain‐specific analyses showed that VCI patients benefited across attention, memory, and executive function, whereas SSD patients primarily improved in memory and executive domains. While sensitivity analyses and publication bias adjustments slightly attenuated effect sizes, the overall conclusions remained stable. Notably, the GRADE assessment revealed a paucity of high‐quality evidence (1.9%), with 40.7% of outcomes rated as very low quality, largely due to inconsistency (substantial unexplained heterogeneity, *I* ^2^ > 50%), risk of bias (methodological limitations including lack of protocol registration and inadequate assessment of primary study bias), and imprecision (wide confidence intervals crossing the null). Overlap across reviews was low (CCA = 4.08%).

**Conclusion:**

HF‐rTMS appears to provide consistent cognitive benefits in Alzheimer's disease and VCI, whereas its efficacy in other neuropsychiatric disorders remains uncertain. Given the low certainty of evidence and substantial heterogeneity, findings should be interpreted with caution. Future large‐scale, multicenter randomized controlled trials with standardized protocols are needed to confirm efficacy and support clinical implementation.

## Graphical abstract

This umbrella review of 29 meta‐analyses found that HF‐rTMS significantly improves cognition in Alzheimer's disease and vascular cognitive impairment. However, GRADE shows that most evidence is of low‐to‐very‐low certainty, and AMSTAR‐2 rates over 60% of reviews as critically low quality. Well‐designed trials are urgently needed.

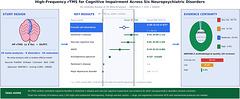

## 1 Introduction

Cognitive impairment is a common and clinically significant feature across a wide range of neuropsychiatric disorders. In the realm of neurodegeneration, it is a hallmark of Alzheimer's disease (AD) (Jack et al. 2024) and a frequent and impactful non‐motor symptom in Parkinson's disease (PD) (Aarsland et al. 2017). In vascular pathology, it presents as vascular cognitive impairment (VCI) (Iadecola et al. 2019). In psychiatric conditions, cognitive deficits are also widely observed in schizophrenia spectrum disorders (SSD) (Green 2006)^,^ major depressive disorder (MDD) (Rhee et al. 2024), and attention‐deficit/hyperactivity disorder (ADHD) (Faraone et al. 2021). These impairments are often persistent rather than transient, contributing to functional decline, reduced quality of life, and poorer long‐term outcomes. As a result, cognitive impairment imposes a substantial burden on patients, caregivers, and healthcare systems (Livingston et al. 2020). Despite this, currently available pharmacological treatments generally provide only modest benefits and may be associated with adverse effects, highlighting the need for safe and effective non‐pharmacological interventions (Livingston et al. 2024).

High‐frequency repetitive transcranial magnetic stimulation (HF‐rTMS) has emerged as a potent candidate in this therapeutic void. By delivering rapid magnetic pulses, HF‐rTMS is thought to induce long‐term potentiation (LTP)‐like plasticity, particularly when targeting the dorsolateral prefrontal cortex (DLPFC). This modulation aims to restore hypoactive neural circuits, theoretically enhancing executive function, working memory, and attention (Lefaucheur et al. 2020). While the DLPFC remains the canonical target, the field remains characterized by substantial heterogeneity in stimulation parameters (e.g., frequency, intensity, and coil positioning), which complicates standardization. Over the last decade, this uncertainty has spawned a deluge of randomized controlled trials (RCTs) and subsequent meta‐analyses, each attempting to pin down the efficacy of rTMS across these diverse pathologies (Hyde et al. 2022).

Several systematic reviews have examined the effects of rTMS on cognitive outcomes across multiple neuropsychiatric conditions, suggesting that HF‐rTMS may improve selected cognitive domains while emphasizing substantial variability in treatment effects across diseases and stimulation protocols (Martin et al. 2016; Zhang et al. 2025). More recent umbrella evidence has further highlighted that the certainty of available evidence remains limited due to methodological shortcomings and substantial heterogeneity (Wu et al. 2025). However, most published reviews either focused on individual disorders or synthesized randomized controlled trials without comprehensively evaluating the methodological quality, overlap of evidence, and certainty of evidence across existing meta‐analyses. Although cross‐disorder evidence is available—including reviews that combined rTMS with psychological interventions (Xu et al. 2023)—important uncertainties remain regarding the robustness and comparative strength of the current evidence base. Furthermore, although shared neurobiological mechanisms—including disrupted large‐scale brain networks and impaired neuroplasticity—have been proposed across several neuropsychiatric disorders, the extent to which these common mechanisms translate into comparable therapeutic responses to HF‐rTMS remains unclear. Thus, there is an urgent need for an umbrella review that rigorously appraises the quality of this evidence to separate signal from noise.

The present study focuses on six major neuropsychiatric conditions, which were selected because they represent the disorders with the most substantial body of published systematic reviews and meta‐analyses evaluating the effects of HF‐rTMS on cognitive outcomes, thereby enabling meaningful cross‐disorder comparisons. Although other neurological or psychiatric disorders have also been investigated, the currently available evidence for those conditions remains insufficient to support a rigorous umbrella review. While this selection is not exhaustive and excludes disorders with limited data (e.g., bipolar disorder, traumatic brain injury), it reflects areas with sufficient data density to support rigorous comparative evaluation. We conducted an umbrella review to synthesize the available evidence, going beyond simple effect sizes to quantitatively explore differences across specific cognitive domains. Crucially, we applied the AMSTAR‐2 tool and the GRADE framework to assess methodological quality and grade the certainty of the evidence. By mapping the strengths and fragility of the current literature, this study aims to provide a robust evidence base to guide clinical decision‐making and steer future research away from redundancy and toward high‐quality, definitive trials.

## 2 Methods

### 2.1 Protocol and Registration

The study protocol was registered in the International Prospective Register of Systematic Reviews (PROSPERO) (registration number: CRD420261351257). The review was conducted and reported in accordance with the *PRISMA 2020 Statement* (adapted for umbrella reviews) (Page et al. 2021) and the methodological guidance from the Joanna Briggs Institute (JBI) Manual for Evidence Synthesis (Aromataris et al. 2015). No substantive modifications to the primary outcomes or eligibility criteria were made compared with the registered protocol. A completed PRISMA 2020 checklist (27 items) is provided in Table S1.

### 2.2 Eligibility Criteria

Strict inclusion and exclusion criteria were defined based on the PICOS‐R (Population, Intervention, Comparator, Outcomes, Study design, Review type) framework.

#### 2.2.1 Study Design

Only published systematic reviews or meta‐analyses were included, and the primary studies within these reviews were predominantly randomized controlled trials (RCTs).

#### 2.2.2 Participants

Adult patients (≥18 years) diagnosed with any of the following conditions were included: (1) Alzheimer's disease (AD) or amnestic mild cognitive impairment (aMCI, considered prodromal AD); (2) vascular cognitive impairment (VCI); (3) schizophrenia spectrum disorders (SSD); (4) major depressive disorder (MDD); (5) attention‐deficit/hyperactivity disorder (ADHD); and (6) Parkinson's disease (PD) with cognitive impairment. These disorders emerged from the systematic literature search as the neuropsychiatric conditions with the largest and most representative body of eligible systematic reviews and meta‐analyses evaluating the effects of HF‐rTMS on cognitive outcomes. Accordingly, they constituted the evidence base for this umbrella review. Although other neuropsychiatric disorders were identified during the search process, the available evidence was too limited or insufficiently synthesized to support meaningful umbrella‐level comparisons.

#### 2.2.3 Intervention/Comparator

The intervention group received high‐frequency repetitive transcranial magnetic stimulation (HF‐rTMS, ≥5 Hz), with or without standard treatment. The control group received sham rTMS combined with the same standard treatment.

#### 2.2.4 Outcomes

Eligible studies were required to report standardized effect sizes for cognitive outcomes—SMD (standardized mean difference), WMD (weighted mean difference), or MD (mean difference)—with corresponding 95% CIs. Data on specific cognitive domains (e.g., executive function, working memory, attention, memory, and processing speed) were preferentially extracted. When domain‐specific outcomes were unavailable, global cognitive scales were extracted as supplementary outcomes.

#### 2.2.5 Exclusion Criteria

No language restrictions were applied during the search. During full‐text screening, only publications in English or Chinese were included, as these were the languages in which all eligible studies were available and could be reliably assessed by the review team. The included studies from Australia, the United Kingdom, and the United States were all published in English peer‐reviewed journals, consistent with this criterion. Conference abstracts, studies without accessible full texts, and animal studies were excluded.

To avoid the loss of evidence within specific disease domains, studies were not excluded solely based on low AMSTAR‐2 ratings. Instead, a strategy of “inclusive selection with stratified interpretation” was adopted, whereby methodological quality was incorporated into GRADE assessment and interpretation of findings.

### 2.3 Search Strategy and Selection Process

CNKI, Cochrane Library, Embase, PubMed, and Web of Science were systematically searched from inception to March 1, 2026. Scopus was not searched because it substantially overlaps with both Embase and Web of Science in biomedical indexing. Previous methodological evaluations have shown that the combined use of PubMed, Embase, and Web of Science provides near‐complete coverage of biomedical systematic reviews. Given the umbrella‐review design and the high degree of database redundancy, inclusion of Scopus was considered unlikely to identify additional eligible reviews while substantially increasing duplicate screening workload. The search strategy combined Medical Subject Headings (MeSH) and free‐text terms, including “transcranial magnetic stimulation,” “cognitive dysfunction,” “systematic review,” “meta‐analysis,” and the names of target disorders. The complete search strategy for PubMed is provided in Table S2.

All records were imported into EndNote 20 for deduplication. Two reviewers independently screened titles and abstracts, followed by full‐text assessment. Discrepancies were resolved through discussion or adjudication by a third reviewer.

### 2.4 Data Extraction

Data extraction was independently performed by two trained reviewers. Before formal extraction, a pilot calibration was conducted using five randomly selected studies to ensure consistency. All extracted data were cross‐checked, and disagreements were resolved through discussion or consultation with a senior reviewer.

The following information was extracted: basic study characteristics, sample size, participant characteristics, outcome measures, statistical results (effect sizes, 95% CIs, *I* ^2^, and publication bias assessments), and methodological details.

Information on stimulation targets (e.g., DLPFC and other cortical regions) was extracted and summarized. Due to inconsistent reporting across reviews, stimulation parameters were not quantitatively synthesized but were considered qualitatively in the interpretation of findings.

The corrected covered area (CCA) was calculated following the method proposed by Pieper et al. (2014) to quantify the degree of overlap of primary studies across systematic reviews.

This metric is defined as CCA = (*Nr* − *Nc*)/[*Nc* × (*Ns* − 1)], where *Nr* is the total number of included study occurrences, *Nc* is the number of unique primary studies, and *Ns* is the number of included systematic reviews; values <5% indicate slight overlap, 5%–10% moderate overlap, and >10% high overlap. According to the predefined decision rule, if the CCA indicated only slight overlap (<5%), no further statistical adjustment for overlap would be undertaken, as duplicate primary evidence was considered unlikely to materially influence pooled estimates.

### 2.5 Methodological Quality Assessment (AMSTAR‐2)

The methodological quality of included reviews was evaluated using the AMSTAR‐2 tool (16 items) (Shea et al. 2017). Two reviewers independently performed the assessment, and Cohen's kappa coefficient was calculated to quantify inter‐rater agreement.

Based on the presence of critical and non‐critical weaknesses, studies were categorized into four levels: high, moderate, low, and critically low quality.

Preliminary assessment indicated considerable variability in methodological quality across this field. Excluding low‐quality studies could have substantially reduced the available evidence base; therefore, all studies were retained for qualitative and quantitative synthesis. Identified methodological limitations from AMSTAR‐2 were incorporated into subsequent GRADE assessments, leading to appropriate downgrading of evidence certainty where necessary (Guyatt et al. 2008).

### 2.6 Certainty of Evidence and Data Synthesis

All statistical analyses were conducted using Stata 16.0 (two‐tailed tests, *p* < 0.05 considered statistically significant).

#### 2.6.1 Data Synthesis and Subgroup Analysis

A random‐effects model using the DerSimonian–Laird method was applied to pool standardized effect sizes (SMD/WMD), generating overall and disease‐specific pooled estimates with 95% CIs and heterogeneity statistics (*I* ^2^). Subgroup analyses were conducted hierarchically. First, primary subgroup analyses were stratified by disease type (AD, VCI, PD, SSD, MDD, ADHD), with between‐group differences tested. Second, where data permitted, nested subgroup analyses were performed according to cognitive domains (e.g., executive function, memory, attention, processing speed) to evaluate domain‐specific effects.

#### 2.6.2 Publication Bias Assessment and Adjustment

Publication bias within each disease subgroup was assessed using visual inspection of funnel plots and quantitatively evaluated using Egger's linear regression test. Begg's test was not additionally performed because Egger's regression is generally considered more sensitive for detecting small‐study effects in meta‐analyses involving continuous outcomes. When the number of included studies was insufficient to ensure adequate statistical power, conclusions were primarily based on bias assessments reported in the original reviews.

If significant publication bias was detected (*p* < 0.05) along with funnel plot asymmetry, the trim‐and‐fill method was applied to estimate potentially missing studies and adjust pooled effect sizes, thereby testing the robustness of the findings.

#### 2.6.3 Sensitivity Analysis

Robustness of the results was evaluated using multiple approaches. A leave‐one‐out analysis was performed by sequentially excluding each review to assess the stability of effect sizes and heterogeneity. Outlier analysis was conducted by identifying extreme values in preliminary analyses and re‐pooling data after their exclusion, with comparisons made before and after adjustment. Although low‐quality studies were not excluded, differences in effect size distributions across AMSTAR‐2 quality levels were examined to qualitatively assess the potential influence of methodological limitations. In addition, influential outliers identified during quantitative synthesis were excluded in post hoc sensitivity analyses to evaluate the robustness of pooled estimates.

#### 2.6.4 Certainty of Evidence (GRADE)

The GRADE framework was applied to independently rate the certainty of evidence for each “disease–cognitive domain” pair (high, moderate, low, or very low). Evidence was downgraded based on the following criteria: Risk of bias: downgraded when the majority of contributing evidence originated from reviews rated as “low” or “critically low” by AMSTAR‐2, particularly when these reviews lacked comprehensive bias assessment of primary trials. To operationalize “suspected risk of bias,” we developed a predefined classification scheme before evidence assessment and applied it consistently across all outcomes to minimize subjective judgment. A meta‐analysis was classified as such if it met any of the following: (a) an AMSTAR‐2 rating of low or critically low due to key methodological limitations (e.g., incomplete search strategy, lack of protocol registration, or inadequate risk‐of‐bias assessment); (b) absence of reported conflicts of interest or funding sources; or (c) inclusion of primary studies with high or unclear risk of bias without sensitivity analyses. Outcomes were downgraded when more than 50% of the contributing evidence was derived from such reviews. The term “suspected” reflects the presence of methodological concerns alongside uncertainty due to incomplete reporting. Inconsistency: downgraded when *I* ^2^ > 50% with inconsistent effect directions, or when substantial unexplained heterogeneity was present (*I* ^2^ > 75%); other factors included indirectness, imprecision (wide CIs or those crossing the null), and uncorrected publication bias.

## 3 Results

### 3.1 Study Selection and Characteristics

**FIGURE 1:**
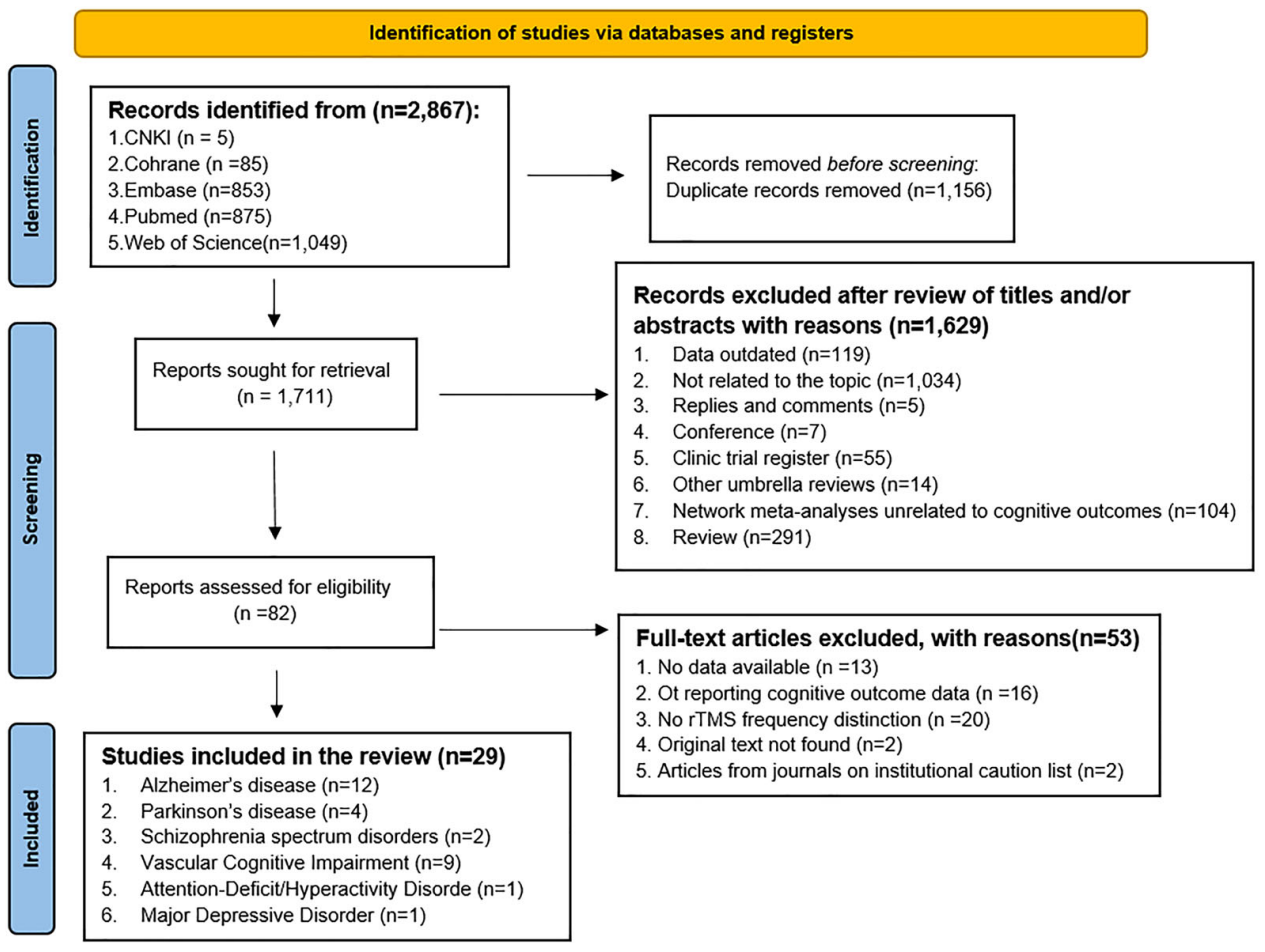
PRISMA flow diagram of the study selection process.

A total of 2867 records were initially identified, and 29 eligible reviews were ultimately included after rigorous screening, comprising 29 systematic reviews with meta‐analyses. The included studies were published between 2015 and 2026 and covered six neuropsychiatric disorders: AD, *n* = 12, VCI, *n* = 9, PD, *n* = 4, SSD, *n* = 2, ADHD, *n* = 1, and MDD, *n* = 1. The study selection process is summarized in the PRISMA flow diagram (Figure 1)

Most studies were conducted in China (*n* = 22), with the remainder originating from Europe, North America, and the Asia–Pacific region. The number of primary RCTs included in each meta‐analysis ranged from 3 to 61, with total sample sizes varying from 74 to 2855 participants. Commonly used cognitive assessment tools included the Mini‐Mental State Examination (MMSE), Montreal Cognitive Assessment (MoCA), Alzheimer's Disease Assessment Scale–Cognitive Subscale (ADAS‐Cog), and Trail Making Test (TMT). Regarding stimulation targets, the dorsolateral prefrontal cortex (DLPFC) was the most frequently targeted region, appearing in 26 of 29 meta‐analyses (89.7%). Other targeted regions included the primary motor cortex, precuneus, parietal/temporal lobes, and multi‐site protocols (Table 1).

**TABLE 1:** Characteristics of the included umbrella reviews/meta‐analyses.

Meta‐analysis (author, year)	Country	Disease category	No. of primary	Total cases	Study design	Analysis model	Key assessment tools	Target locations
Hsu et al. (2015)	USA	AD	6	152	RCTs	Random effects	MMSE	DLPFC, temporoparietal network
Lawrence et al. (2017)	Australia	PD	3	74	RCTs	Random effects	FAB, TMT‐A	DLPFC, motor cortices
Jiang et al. (2019)	China	SSD	9	351	RCTs	Fixed effects	WCST, TOL, TMT‐B, and others	DLPFC
Jiang et al. (2021)	China	AD & MCI	8	164	RCTs	Random effects	MoCA, MMSE, RBMT, CMS, TMT‐A/B	DLPFC
Jiang et al. (2020)	China	PD	11	320	RCTs	Fixed effects	DRS, MMSE, MoCA	DLPFC
Wang et al. (2020)	China	AD	10	166	RCTs	Fixed effects	MMSE, ADAS‐cog, TMT	Bilateral DLPFC; Broca's/Wernicke's areas; parietal areas; temporal areas
Wang et al. (2021)	China	AD & BPSD	20	1156	RCTs	Fixed effects	MMSE, ADAS‐cog, BEHAVE‐AD	DLPFC
Teselink et al. (2021)	Canada	AD & BPSD	13	380	RCTs	Random effects	MMSE, MoCA, ADAS‐cog, GDS, AES, BEHAVE‐AD	DLPFC, precuneus, parietal/temporal lobes
Wang et al. (2021)	China	AD & MCI	14	215	RCTs	Fixed or random effects	MMSE, MoCA	DLPFC, precuneus, parietal
He et al. (2022)	China	PD	12	215	RCTs	Random effects	MMSE, MoCA	DLPFC, primary motor cortex, inferior frontal gyrus, premotor cortex
Šimko et al. (2022)	Czechia	AD	16	415	RCTs	Random effects	MMSE, ADAS‐cog, 3MS, MoCA	DLPFC, precuneus, parietal/temporal regions
Xu et al. (2022)	China	VCI (PSCI)	9	541	RCTs	Random effects	MoCA, RBMT, LOTCA, DS, CAMPROMPT, MBI, P300	DLPFC; prefrontal cortex; posterior temporal cortex
Zhang et al. (2022)	China	AD	8	329	RCTs	Fixed effects	MMSE, ADAS‐cog	DLPFC; multi‐sites (neuro‐AD protocol: Broca's, Wernicke's, pSAC); left lateral parietal cortex; left lateral temporal lobe; parietal–occipital junction
Chen et al. (2023)	China	VCI (PSCI)	41	2855	RCTs	Random effects	MMSE, MoCA, NCSE	DLPFC; multi‐sites (frontal, temporal, occipital lobes)
Chen et al. (2023)	China (Taiwan)	ADHD	5	189	RCTs	Random effects	DSM‐5 criteria	Prefrontal cortex
Gong et al. (2023)	China	VCI (PSCI)	6	182	RCTs	Fixed effects	MMSE, MoCA, LOTCA	DLPFC
Miller et al. (2023)	UK	AD	16	244	RCTs	Fixed effects	MMSE, MoCA	DLPFC
Chen et al. (2024)	China	VCI (PSCI)	61	2849	RCTs	Random effects	MMSE, MoCA, LOTCA, P300	DLPFC, frontal lobe/temporal lobe/ occipital lobe
Le et al. (2024)	Vietnam	VCI (PSCI)	10	496	RCTs	Random effects	MMSE, MoCA	DLPFC
Wang et al. (2024)	China	SSD	35	810	RCTs	Random effects	PANSS, BPRS, SANS	Prefrontal cortex
Wang et al. (2024)	China	VCI	19	1016	RCTs	Fixed effects	MMSE, MoCA, OCS, TMT, CDT, BADS	DLPFC
Xiu et al. (2024)	Italy	AD	17	631	RCTs	Fixed effects	MMSE, MoCA, ADAS‐cog, P300	DLPFC
Zhao et al. (2024)	China	VCI (PSCI)	11	342	RCTs	Random effects	MMSE, MBI, MoCA, LOTCA, RBANS, K‐MMSE, K‐MoCA	DLPFC
Zhu et al. (2024)	China	VCI (PSCI)	6	182	RCTs	Random effects	MMSE, MoCA, LOTCA, MBI, RBANS, RBMT	DLPFC
Fu et al. (2025)	China	MDD	36	784	RCTs	Random effects	DSM‐5, DSM‐4, ICD‐10 criteria	DLPFC; left dorsomedial PFC; left middle frontal gyrus
Hou et al. (2025)	China	AD	24	648	RCTs	Fixed effects	MMSE, MoCA, CDR, HIS, BEHAVE‐AD, ADLs	DLPFC; lateral temporal lobe; prefrontal cortex; parietal lobe; precuneus; left hippocampus
Wang et al. (2025)	China	AD & MCI	31	1363	RCTs	Random effects	MMSE, MoCA, ADAS‐cog	DLPFC; left parietal lobe; angular gyrus; cerebellar vermis; parietal–hippocampal; precuneus
Zhao et al. (2025)	China	VCI (PSCI)	7	279	RCTs	Random effects	MMSE, MoCA, MBI, LOTCA, RBANS	DLPFC, primary motor cortex
Lu et al. (2026)	China	PD	32	727	RCTs	Random effects	MoCA, UPDRS‐III, BDI, FOG‐Q	DLPFC, primary motor cortex; supplementary motor area

*Note*: DSM/ICD refers to diagnostic criteria manuals. Analysis model: “Mixed” indicates the use of both fixed‐ and random‐effects models within the study.

Abbreviations: Study design: RCTs, randomized controlled trials. Disease categories: AD, Alzheimer's disease; MCI, mild cognitive impairment; BPSD, behavioral and psychological symptoms of dementia; PD, Parkinson's disease; SSD, schizophrenia spectrum disorders; VCI, vascular cognitive impairment; PSCI, post‐stroke cognitive impairment; ADHD, attention‐deficit/hyperactivity disorder; MDD, major depressive disorder; DLPFC, dorsolateral prefrontal cortex. Assessment Tools: MMSE, Mini‐Mental State Examination; MoCA, Montreal Cognitive Assessment; ADAS‐cog, Alzheimer's Disease Assessment Scale–Cognitive Subscale; TMT, Trail Making Test; WCST, Wisconsin Card Sorting Test; FAB, Frontal Assessment Battery; TOL, Tower of London; RBMT, Riverweed Behavioral Memory Test; RBANS, Repeatable Battery for the Assessment of Neuropsychological Status; LOTCA, Loewenstein Occupational Therapy Cognitive Assessment; MBI, Memory Box Index; DS, Digit Span; CAMPROMPT, Cambridge Prospective Memory Test; OCS, Oxford Cognitive Screen; CDT, Clock Drawing Test; BADS, Behavioral Assessment of the Dysexecutive Syndrome; NCSE, Neurobehavioral Cognitive Status Examination; DRS, Dementia Rating Scale; 3MS, Modified Mini‐Mental State Examination; K‐MMSE/K‐MoCA, Korean versions; CDR, Clinical Dementia Rating; BEHAVE‐AD, Behavioral Pathology in Alzheimer's Disease Rating Scale; PANSS, Positive and Negative Syndrome Scale; BPRS, Brief Psychiatric Rating Scale; SANS, Scale for the Assessment of Negative Symptoms; AES, Apathy Evaluation Scale; GDS, Geriatric Depression Scale (or Global Deterioration Scale, depending on context); ADLs, Activities of Daily Living; HIS, Hachinski Ischemic Score; UPDRS‐III, Unified Parkinson's Disease Rating Scale Part III; BDI, Beck Depression Inventory; FOG‐Q, Freezing of Gait Questionnaire; P300, P300 Event‐Related Potential.

**FIGURE 2:**
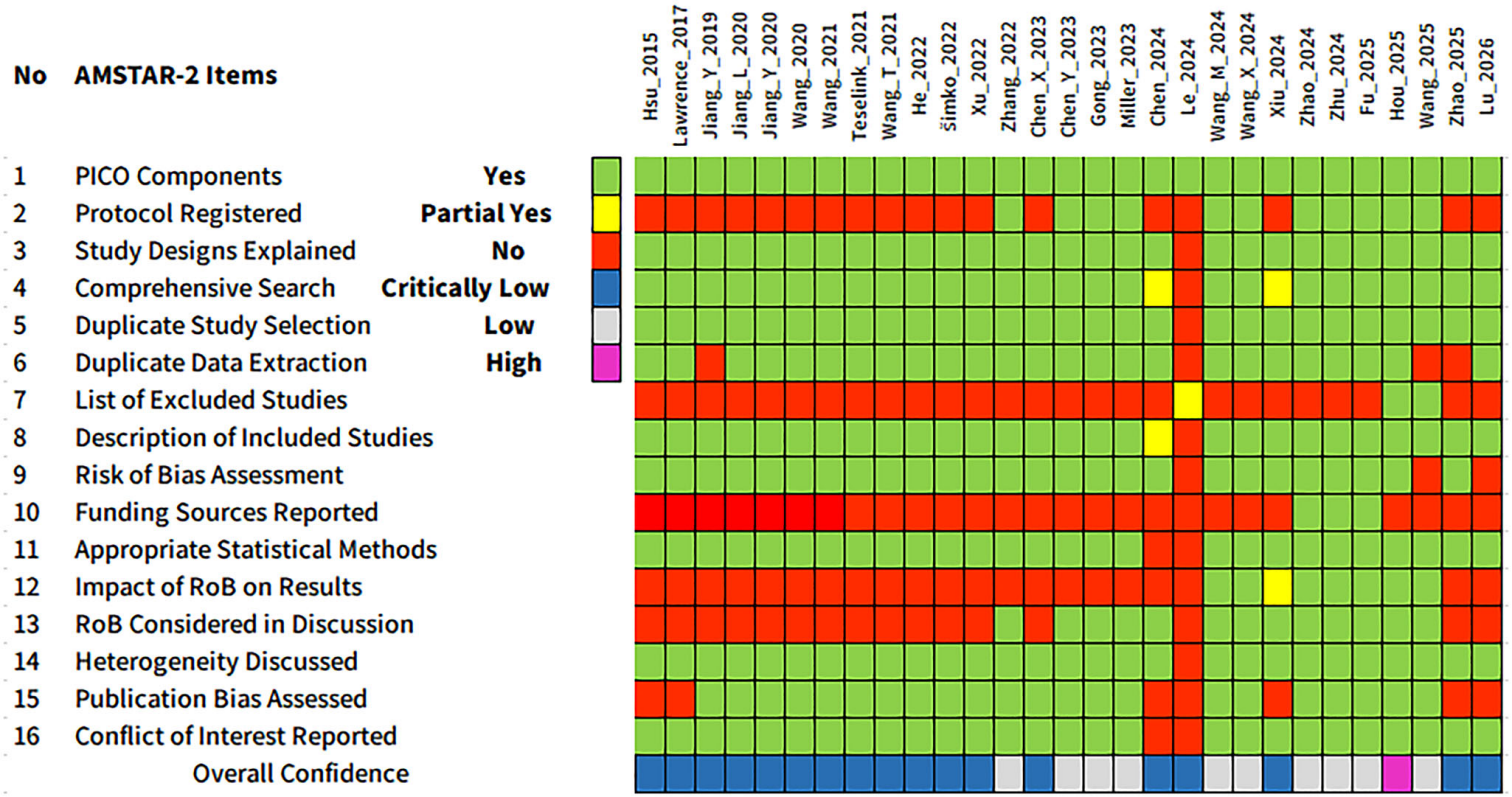
Methodological quality assessment of included meta‐analyses using the AMSTAR‐2 tool.

AMSTAR‐2 assessment indicated generally poor methodological quality: only one study (3.4%) was rated as high quality, 10 studies (34.5%) as low quality, and 18 studies (62.1%) as critically low quality (Figure 2). The main limitations included a lack of protocol registration, insufficient assessment of risk of bias in primary studies, and the absence of conflict‐of‐interest statements. Within the AMSTAR‐2 framework, protocol registration (Item 2) is a critical domain because pre‐registration mitigates selective outcome reporting; its absence raises concerns about reporting bias. Inadequate risk‐of‐bias assessment (Item 9, also critical) means reviews may propagate uncontrolled confounds from primary trials. Conflict‐of‐interest disclosure (Item 16), while non‐critical, affects credibility. The combination of these limitations drove the high proportion of critically low ratings.

### 3.2 Overlap of Included Reviews (CCA)

The degree of overlap among the included meta‐analyses was quantified using the CCA. The calculated CCA was 4.08%, indicating a slight degree of overlap (<5%). Of the 29 included meta‐analyses, 26 provided sufficient primary study reference lists to calculate overlap; the remaining three (Jiang et al. 2020; Jiang et al. 2021; and Wang et al. 2021) lacked DOI numbers and the corresponding authors did not respond to requests for primary study lists, so they were excluded from the overlap analysis. Detailed overlap information is provided in Table S3. Given this low overlap, the included meta‐analyses were considered sufficiently independent to support the subsequent quantitative re‐meta‐analysis, although residual dependence could not be entirely excluded and was considered when interpreting the findings.

### 3.3 Overall Effects and Disease‐Specific Subgroup Analyses

**FIGURE 3:**
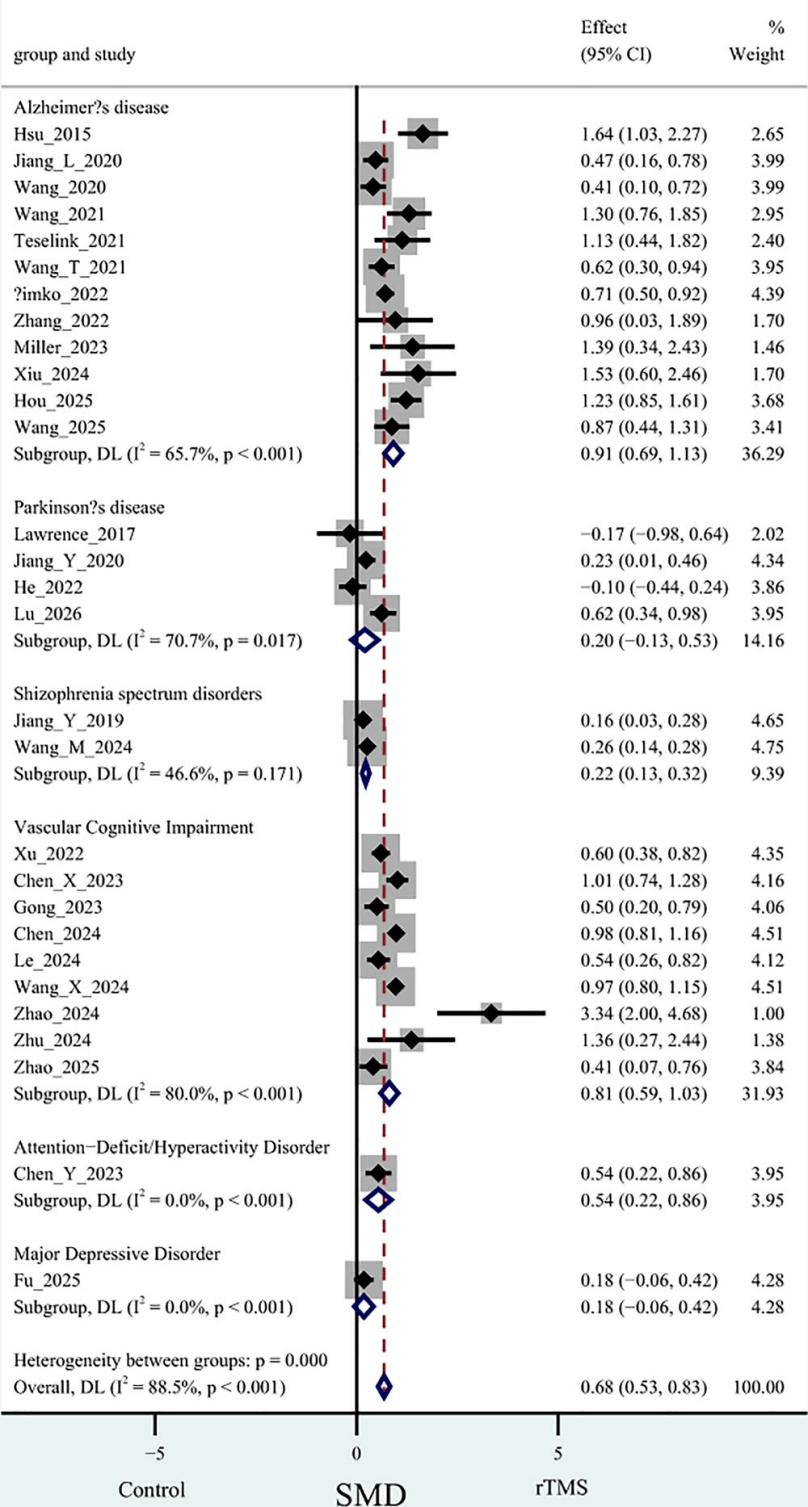
Forest plot of the efficacy of high‐frequency rTMS on cognitive function across six neuropsychiatric disorders (initial analysis including all studies). This figure presents the pooled standardized mean differences (SMDs) with 95% confidence intervals for each disease subgroup before any sensitivity or bias adjustment. Note the extreme outlier in the Vascular Cognitive Impairment group (Zhao et al. 2024, SMD = 3.34), which prompted further robustness checks.

The overall pooled analysis of all eligible meta‐analytic evidence across the included disorders showed a moderate‐to‐large effect (SMD = 0.68, 95% CI: 0.53–0.83, *p* < 0.001), although substantial heterogeneity was observed (*I* ^2^ = 88.5%; Figure 3). Subgroup analyses demonstrated that this significant overall pooled effect reflected the combined evidence across all included disorders rather than consistent benefits within every disease subgroup. Significant improvements were observed in AD, VCI, ADHD, and SSD, whereas no statistically significant improvements were identified in PD. For MDD, only one eligible meta‐analysis was available, precluding robust conclusions regarding treatment efficacy (test for subgroup differences, *p* < 0.001).

Among the disease‐specific subgroups, the largest effect size was observed in AD (SMD = 0.91, 95% CI: 0.69–1.13), followed by VCI (SMD = 0.81, 95% CI: 0.59–1.03). ADHD (SMD = 0.54) and SSD (SMD = 0.22) showed moderate‐to‐small but statistically significant effects, whereas no statistically significant improvements were identified in PD. For MDD, only one meta‐analysis met the inclusion criteria (Fu et al. 2025), which reported a null effect. Given the limited evidence base, the conclusion for MDD should be considered preliminary and inconclusive, rather than definitive. Notably, one study in the VCI subgroup (Zhao et al. 2024) reported an extremely large effect size (SMD = 3.34) with a relatively small weight (2.29%), which may reflect the influence of small sample size or short‐term, domain‐specific cognitive measures. Therefore, this unusually large effect is more likely attributable to study‐specific methodological characteristics than to a true treatment effect and should be interpreted with caution.

To evaluate the impact of this outlier and potential publication bias, additional robustness analyses were conducted. First, after excluding Zhao et al. (2024), the pooled effect size for VCI decreased to SMD = 0.75 (95% CI: 0.57–0.94), and the overall effect size was adjusted to SMD = 0.65 (95% CI: 0.50–0.80), with a slight reduction in heterogeneity (*I* ^2^ = 88.0%), indicating that the main findings remained stable (Figure S1). Second, visual inspection of the funnel plot suggested asymmetry, and Egger's test indicated significant publication bias (*p* < 0.001). After adjustment using the trim‐and‐fill method (with five potentially missing studies imputed), the pooled effect size decreased to SMD = 0.59 (95% CI: 0.44–0.74, *p* < 0.001). Although the effect size was attenuated (approximately 13.7%), it remained at a moderate level, further supporting the robustness of the findings (Figure S2).

### 3.4 Domain‐Specific Subgroup Analyses

**FIGURE 4:**
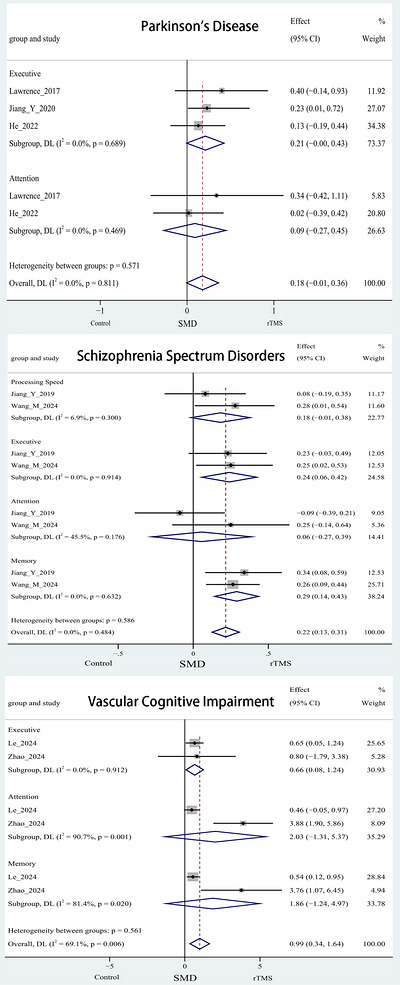
Subgroup analysis of cognitive domains by disease category in included meta‐analyses.

Domain‐specific subgroup analyses were conducted for PD, SSD, and VCI (Figure 4). These three disease groups had sufficient meta‐analytic evidence reporting cognitive outcomes disaggregated by specific cognitive domains. For AD, ADHD, and MDD, the available meta‐analyses predominantly reported only global cognitive scores without domain‐specific data, precluding reliable domain‐level synthesis.

In patients with PD, rTMS showed a borderline significant improvement in executive function (SMD = 0.21, 95% CI: ‐0.01 to 0.43), with no heterogeneity within this subgroup (*I* ^2^ = 0%). In contrast, no statistically significant improvement was observed in attention (SMD = 0.09, 95% CI: ‐0.27 to 0.45). Differences across cognitive domains were not statistically significant (*p* = 0.571).

In patients with SSD, differential effects were observed across cognitive domains. Significant improvements were found in memory (SMD = 0.29, 95% CI: 0.14–0.43) and executive function (SMD = 0.24, 95% CI: 0.06–0.42), both with no heterogeneity (*I* ^2^ = 0%). Processing speed showed a borderline effect (SMD = 0.18, 95% CI: ‐0.01 to 0.37), while no significant improvement was observed in attention (SMD = 0.04, 95% CI: ‐0.20 to 0.27). Although trends varied across domains, between‐domain differences were not statistically significant (*p* = 0.342).

In patients with VCI, rTMS demonstrated significant improvements across multiple cognitive domains, albeit with substantial heterogeneity. The largest effect was observed for attention (SMD = 0.67, 95% CI: 0.18–1.17), accompanied by considerable heterogeneity (*I* ^2^ = 90.7%, *p* = 0.001). Significant improvements were also observed in memory (SMD = 0.61, 95% CI: 0.20–1.03) and executive function (SMD = 0.66, 95% CI: 0.08–1.24), with the latter showing no heterogeneity (*I* ^2^ = 0%). Overall, significant improvements were observed across multiple cognitive domains in VCI. Although effect sizes varied numerically, no statistically significant differences between cognitive domains were detected (*p* = 0.983), suggesting broadly comparable treatment effects across the reported domains.

### 3.5 Certainty of Evidence (GRADE)

The certainty of evidence for 54 specific outcomes was assessed using the GRADE framework (Table 2). Overall, the quality of evidence was low: only one outcome (1.9%) was rated as high quality; 14 outcomes (25.9%) as moderate quality; 17 outcomes (31.5%) as low quality; and the remaining 22 outcomes (40.7%) as very low quality.

**TABLE 2:** GRADE evidence certainty assessment for each included meta‐analysis outcome.

Author, year	Outcome domain	Effect estimate (95% CI)	Measure	Heterogeneity (*I^2^*)	Pub. Bias (*P*)	Certainty rating	Reasons for downgrading
Hsu et al. (2015)	Global cognition	1.64 (1.03 to 2.27)	SMD	79.60%	0.78	⨁◯◯◯ Very Low	Very serious inconsistency; suspected risk of bias
Lawrence et al. (2017)	Global cognition	−0.17 (–0.98 to 0.64)	SMD	NR	NR	⨁◯◯◯ Very Low	Imprecision; suspected risk of bias
	Executive function	0.40 (–0.14 to 0.93)	SMD	0%	0.92	⨁◯◯◯ Very Low	Imprecision; suspected risk of bias
	Attention	0.34 (–0.42 to 1.11)	SMD	NR	NR	⨁◯◯◯ Very Low	Imprecision; suspected risk of bias
Jiang et al. (2019)	Global cognition	0.16 (0.03 to 0.28)	SMD	0%	NA	⨁⨁⨁◯ Moderate	Suspected risk of bias only
	Processing speed	0.08 (–0.19 to 0.35)	SMD	24%	NA	⨁⨁◯◯ Low	Imprecision; suspected risk of bias
	Executive function	0.23 (–0.03 to 0.49)	SMD	0%	NA	⨁⨁◯◯ Low	Imprecision; suspected risk of bias
	Attention	−0.09 (–0.39 to 0.21)	SMD	0%	NA	⨁⨁◯◯ Low	Imprecision; suspected risk of bias
	Memory	0.34 (0.08 to 0.59)	SMD	0%	NA	⨁⨁⨁◯ Moderate	Suspected risk of bias only
	Language	0.14 (–0.18 to 0.45)	SMD	0%	NA	⨁⨁◯◯ Low	Imprecision; suspected risk of bias
Jiang et al. (2021)	Global cognition	0.47 (0.16 to 0.78)	SMD	40%	NA	⨁⨁◯◯ Low	Original review rated as Low
Jiang et al. (2020)	Executive function	0.23 (0.01 to 0.46)	SMD	0%	NA	⨁⨁⨁◯ Moderate	Suspected risk of bias only
Wang et al. (2020)	Global cognition	0.41 (0.10 to 0.72)	SMD	0%	0.97	⨁⨁⨁◯ Moderate	Suspected risk of bias only
Wang et al. (2021)	Global cognition	1.30 (0.76 to 1.85)	SMD	93%	NA	⨁◯◯◯ Very Low	Very serious inconsistency
Teselink et al. (2021)	Global cognition	1.13 (0.44 to 1.82)	SMD	87.40%	>0.05	⨁⨁◯◯ Low	Very serious inconsistency
Wang et al. (2021)	Executive function	0.55 (0.20 to 0.90)	SMD	0%	NA	⨁⨁⨁◯ Moderate	Suspected risk of bias only
	Memory	0.94 (0.19 to 1.69)	SMD	84%	NA	⨁◯◯◯ Very Low	Very serious inconsistency; suspected risk of bias
He et al. (2022)	Global cognition	−0.10 (–0.44 to 0.24)	SMD	36.70%	NR	⨁◯◯◯ Very Low	Imprecision; suspected risk of bias
	Executive function	0.13 (–0.19 to 0.44)	SMD	0%	0.13	⨁◯◯◯ Very Low	Imprecision; suspected risk of bias
	Attention & memory	0.02 (–0.39 to 0.42)	SMD	0%	NR	⨁◯◯◯ Very Low	Imprecision; suspected risk of bias
Šimko et al. (2022)	Global cognition	0.71 (0.50 to 0.92)	SMD	18.90%	0.57	⨁⨁⨁◯ Moderate	Suspected risk of bias only
Xu et al. (2022)	Memory	1.92 (1.21 to 2.62)	WMD	29%	NA	⨁⨁⨁◯ Moderate	Suspected risk of bias only
Zhang et al. (2022)	Global Cognition	1.87 (1.59 to 2.15)	WMD	0%	>0.05	⨁⨁⨁⨁ High	No serious limitations
Chen et al. (2023)	ADLs	9.38 (7.19 to 11.58)	WMD	83%	NA	⨁◯◯◯ Very Low	Very serious inconsistency; suspected risk of bias
Chen et al. (2023)	Global cognition	0.54 (0.22 to 0.86)	SMD	0%	NA	⨁⨁◯◯ Low	Suspected risk of bias; imprecision
	Processing speed	0.59 (0.22 to 0.96)	SMD	0%	NA	⨁⨁◯◯ Low	Suspected risk of bias; imprecision
	Memory	0.18 (–0.52 to 0.87)	SMD	71%	NA	⨁◯◯◯ Very Low	Very serious inconsistency; suspected risk of bias
	Executive function	0.23 (–0.24 to 0.71)	SMD	40%	NA	⨁◯◯◯ Very Low	Imprecision; suspected risk of bias
Gong et al. (2023)	Global cognition	2.41 (1.13 to 3.69)	WMD	0%	NA	⨁⨁⨁◯ Moderate	Original review rated as Moderate
Miller et al. (2023)	Global cognition	1.39 (0.34 to 2.43)	SMD	85.80%	NA	⨁◯◯◯ Very Low	Very serious inconsistency; suspected risk of bias
Chen et al. (2024)	Global cognition	2.44 (1.74 to 3.13)	WMD	51%	NA	⨁⨁◯◯ Low	Serious inconsistency; suspected risk of bias
Le et al. (2024)	Executive function	0.65 (0.05 to 1.24)	SMD	93%	NA	⨁◯◯◯ Very Low	Very serious inconsistency
	Attention	0.46 (–0.05 to 0.97)	SMD	64%	NA	⨁◯◯◯ Very Low	Very serious inconsistency
	Memory	0.54 (0.12 to 0.95)	SMD	88%	NA	⨁◯◯◯ Very Low	Very serious inconsistency
Wang et al. (2024)	Executive function	0.25 (0.02 to 0.53)	SMD	0%	0.69	⨁⨁⨁◯ Moderate	Suspected risk of bias only
	Memory	0.26 (0.09 to 0.44)	SMD	49.90%	NA	⨁⨁⨁◯ Moderate	Suspected risk of bias only
	Attention	0.25 (–0.14 to 0.64)	SMD	63.30%	NA	⨁◯◯◯ Very Low	Inconsistency; imprecision; suspected risk of bias
	Processing speed	0.28 (0.01 to 0.54)	SMD	0%	NA	⨁⨁⨁◯ Moderate	Suspected risk of bias only
Wang et al. (2024)	Executive function	0.97 (0.80 to 1.15)	SMD	0%	0.64	⨁⨁⨁◯ Moderate	Original review rated as Moderate
Xiu et al. (2024)	Global cognition	3.64 (1.86 to 5.42)	WMD	95%	NA	⨁◯◯◯ Very Low	Very serious inconsistency; suspected risk of bias
Zhao et al. (2024)	Global cognition	3.34 (2.00 to 4.68)	SMD	93.70%	0.43	⨁⨁◯◯ Low	Very serious inconsistency
	Attention	3.88 (1.90 to 5.86)	SMD	>50%	0.02	⨁◯◯◯ Very Low	Serious inconsistency; Publication bias
	Executive function	0.80 (–1.79 to 3.38)	SMD	<50%	0.62	⨁⨁◯◯ Low	Imprecision; suspected risk of bias
	Language	4.76 (1.22 to 8.31)	SMD	<50%	0.01	⨁⨁◯◯ Low	Publication bias
	Memory	3.76 (1.07 to 6.45)	SMD	>50%	0.01	⨁◯◯◯ Very Low	Serious inconsistency; publication bias
Zhu et al. (2024)	Global cognition	1.36 (0.27 to 2.44)	SMD	88.80%	0.27	⨁◯◯◯ Very Low	Very serious inconsistency; suspected risk of bias
Fu et al. (2025)	Global cognition	0.18 (–0.06 to 0.42)	SMD	0%	NA	⨁⨁◯◯ Low	Imprecision; suspected risk of bias
	Memory	−0.08 (–0.42 to 0.26)	SMD	15%	NA	⨁⨁◯◯ Low	Imprecision; suspected risk of bias
	Processing speed	0.03 (–0.43 to 0.49)	SMD	62%	NA	⨁◯◯◯ Very Low	Inconsistency; imprecision; suspected risk of bias
	Language	0.53 (–0.01 to 1.07)	SMD	0%	NA	⨁⨁◯◯ Low	Imprecision (borderline); suspected risk of bias
Hou et al. (2025)	ADLs	−3.00 (–3.32 to –2.68)	WMD	0%	NA	⨁⨁⨁◯ Moderate	Suspected risk of bias only
Wang et al. (2025)	Global cognition	0.87 (0.44 to 1.31)	SMD	NR	0.25	⨁⨁◯◯ Low	Heterogeneity not reported; suspected risk of bias
Zhao et al. (2025)	Global cognition	0.41 (0.07 to 0.76)	SMD	0%	NA	⨁⨁⨁◯ Moderate	Suspected risk of bias only
Lu et al. (2026)	Motor function	−0.62 (–0.98 to –0.34)	SMD	68%	NA	⨁⨁◯◯ Low	Serious inconsistency; suspected risk of bias

*Note*: Each row represents a specific outcome analysis within an included meta‐analysis. The certainty rating is determined by the lowest score across the five GRADE domains. A single meta‐analysis (e.g., Zhao et al. 2024) may receive different ratings for different outcomes depending on the specific heterogeneity and precision of that outcome.

GRADE certainty levels:

High (⨁⨁⨁⨁): Further research is very unlikely to change our confidence in the estimate of effect.

Moderate (⨁⨁⨁◯): Further research is likely to have an important impact on our confidence in the estimate of effect and may change the estimate.

Low (⨁⨁◯◯): Further research is very likely to have an important impact on our confidence in the estimate of effect and is likely to change the estimate.

Very Low (⨁◯◯◯): We are very uncertain about the estimate.

Reasons for downgrading (GRADE domains):

Risk of bias (or suspected risk of bias): Downgraded if the majority of contributing evidence originated from meta‐analyses with AMSTAR‐2 ratings of “low” or “critically low,” or if the original reviews lacked protocol registration, comprehensive bias assessment, or disclosed conflicts of interest. “Suspected” indicates uncertainty remains but methodological limitations are present.

Inconsistency: Downgraded if heterogeneity was substantial (*I* ^2^ > 50%) or if there was unexplained variability in effect estimates across studies. “Very serious” typically indicates *I* ^2^ > 75% or conflicting directions of effect.

Imprecision: Downgraded if the 95% CI included the null effect (no difference) with a wide range, or if the total sample size/events were below the optimal information size (OIS).

Publication bias: Downgraded if statistical tests (Egger's/Begg's) indicated asymmetry (*p* < 0.05) or if funnel plots suggested missing small studies.

Indirectness (not applied in this table): Downgraded if evidence did not directly address the PICO question.

Abbreviations: ADLs, activities of daily living; CI, confidence interval; GRADE, Grading of Recommendations Assessment, Development and Evaluation; *I* ^2^, heterogeneity index (percentage of variation across studies due to heterogeneity rather than chance); NA, not applicable (e.g., publication bias test not performed due to 10 included studies); NR, not reported; Pub. Bias, publication bias (assessed by Egger's or Begg's test); SMD, standardized mean difference; WMD, weighted mean difference.

The primary reasons for downgrading included risk of bias (affecting 41 outcomes), substantial unexplained heterogeneity (affecting 19 outcomes, including 13 outcomes with *I* ^2^ > 75%), imprecision (affecting 19 outcomes), and publication bias (affecting 3 outcomes). Among these, substantial between‐study heterogeneity was the most frequent contributor to reduced evidence certainty, indicating considerable variability in treatment effects across diseases, stimulation protocols, and cognitive outcome measures. Notably, serious inconsistency (i.e., high heterogeneity) was the predominant driver of evidence downgrading, particularly in key outcomes such as global cognitive function (6 outcomes), memory (2 outcomes), and executive function (1 outcome).

## 4 Discussion

Previous cross‐disorder reviews have generally suggested that HF‐rTMS may improve cognitive performance across several neuropsychiatric disorders. However, these reviews primarily summarized treatment effects without systematically evaluating methodological quality, overlap among reviews, or certainty of evidence. To address these gaps, the present umbrella review synthesizes 29 meta‐analyses published through March 2026 and applies AMSTAR‐2, CCA, and GRADE to appraise the methodological quality, evidence overlap, and certainty of evidence across six neuropsychiatric disorders, complemented by a quantitative re‐meta‐analysis. While the re‐meta‐analysis yields a moderate‐to‐large overall effect size, efficacy varies significantly by disease, and the certainty of evidence for most outcomes remains low to very low. Our findings therefore extend previous work by demonstrating that statistically significant pooled effects do not necessarily translate into high‐certainty evidence for clinical decision‐making.

Subgroup analyses reveal distinct therapeutic patterns driven by pathophysiology. The most robust benefits were observed in AD and VCI, whereas effects in other disorders were smaller or absent. This pattern likely reflects multiple non‐mutually exclusive factors. First, AD and VCI may involve relatively localized network pathology—temporoparietal networks in AD and frontal‐subcortical circuits in VCI—that overlaps more directly with DLPFC‐centered networks modulated by rTMS. However, because the DLPFC is also a common target in other disorders, mechanistic overlap alone is unlikely to fully account for the differential effects; the responsiveness of the targeted networks to neuromodulation may vary across disease states. Second, the evidence base for AD (*n* = 12) and VCI (*n* = 9) is substantially larger than for ADHD (*n* = 1) and MDD (*n* = 1), affording more stable effect estimates and greater power to detect true effects. Conversely, conclusions for ADHD and MDD remain particularly tentative given the limited number of available reviews. Third, outcome measurement varied considerably across disorders. AD studies more consistently used ADAS‐cog—a disease‐validated and widely accepted instrument—whereas other disorders employed more heterogeneous cognitive batteries with variable sensitivity to change and inconsistent psychometric validation. This measurement heterogeneity may have contributed to the imprecision and inconsistency identified in the GRADE assessment. These explanations are speculative and not mutually exclusive; direct head‐to‐head comparative studies with harmonized cognitive batteries and standardized stimulation parameters are needed to disentangle the relative contributions of these factors.

Under the triple‐network model, AD patients show reduced segregation between the DLPFC (a central executive network [CEN] core node) and the DMN, a disruption directly linked to memory and executive deficits (Zhang et al. 2022)^.^ HF‐rTMS may induce LTP‐like effects, enhancing connectivity between cortical regions (e.g., parietal cortex) and the hippocampus to foster synaptic plasticity and associative memory (Wang et al. 2014). In VCI, despite primary white matter pathology, brain networks often reorganize to compensate for structural injury (Morgan and Mc Auley 2024). Observed cognitive gains, particularly in attention, may stem from this compensatory activation. However, substantial heterogeneity and extreme effect sizes in the VCI subgroup suggest that lesion location, severity, and comorbidities act as key modifiers, warranting refined stratified analyses in future studies. Importantly, sensitivity analyses demonstrated that the beneficial effect in VCI remained statistically significant after exclusion of the influential outlier, suggesting that the observed treatment benefit was not solely driven by a single study.

Conversely, SSD shows only small‐to‐moderate effects restricted to memory and executive function, whereas PD shows no significant cognitive improvement. For MDD, only one meta‐analysis met the inclusion criteria, which reported a null effect; therefore, the conclusion for MDD remains inconclusive and requires further investigation. In SSD, widespread neurodevelopmental abnormalities disrupt the whole‐brain structural connectome and topology (Mandal et al. 2022); thus, targeting a single region like the DLPFC may be insufficient to modulate global dysfunction, although this hypothesis requires direct testing in studies with multi‐site stimulation or network‐targeted approaches.

For PD, cognitive impairment involves complex network reorganization, specifically disrupted frontostriatal connectivity and subcortical‐cortical decoupling (Devignes et al. 2022). This long‐range disconnection may partially limit the capacity of standard DLPFC‐targeted HF‐rTMS to effectively modulate subcortical network function (Beheshti and Ko 2021). Furthermore, it remains unclear if current parameters (e.g., frequency, pulse number) can modulate subcortical nuclei via functional connectivity from cortical stimulation sites, rather than through direct physical penetration of the magnetic field, which is limited to cortical depths. The limited number of PD studies also raises the possibility of false‐negative findings due to low statistical power.

The substantial heterogeneity in the overall analysis deserves further discussion. This is expected given the diverse conditions, stimulation protocols, and outcome measures included. We identified three contributory factors. First, clinical heterogeneity across disorders introduces variability in baseline cognitive profiles, disease stages, and underlying pathophysiology. Even within the same disease, meta‐analyses differed in inclusion criteria for cognitive impairment severity (e.g., mild vs. moderate AD). Second, methodological heterogeneity in primary RCTs included varying stimulation parameters: frequency (5 Hz vs. 10 Hz vs. 20 Hz), number of pulses per session, total sessions, whether cognitive training was co‐administered, and target localization methods. Most original meta‐analyses did not conduct subgroup analyses by these parameters, so we could not statistically isolate their individual contributions. Given the central role of DLPFC‐targeted HF‐rTMS in most included studies, the observed effects may primarily reflect modulation of frontoparietal executive networks, whereas evidence for alternative targets remains insufficient for comparative inference. Third, measurement heterogeneity regarding cognitive domains and specific tests used across studies further contributed to the variability.

Methodological quality was poor: over 60% of included studies were rated critically low on AMSTAR‐2 (see Results for detailed item‐level assessment). These methodological shortcomings—particularly the lack of protocol registration and inadequate bias assessment—directly drove the downgrading of evidence certainty in GRADE evaluations across most outcomes. High heterogeneity further reflected variability in participants, stimulation parameters, and controls. Some original meta‐analyses pooled data without exploring these sources, likely inflating effect sizes (Ioannidis 2008).

Sensitivity analyses show that while excluding outliers and adjusting for publication bias attenuates the effect size, it remains statistically significant. This suggests the therapeutic effect is genuine, though likely overestimated by the current low‐quality evidence base.

Unlike single‐disease reviews, this study offers a cross‐disorder comparison within a unified framework. Our findings refine previous conclusions; for instance, while early small studies suggested rTMS benefits in PD, the inclusion of higher‐quality data here no longer supports a significant effect. This underscores the value of umbrella reviews in consolidating fragmented evidence. Additionally, our strategy of inclusive selection with stratified interpretation, paired with GRADE, transparently represents current uncertainties.

Limitations include the lack of individual participant data (IPD), preventing adjustment for covariates like age, disease duration, and medication. Subgroup analyses for conditions like ADHD and MDD were limited by study numbers, potentially affecting statistical stability. Incomplete reporting of intervention parameters (e.g., coil type, localization) in original reviews precluded dose–response analyses. Finally, despite adjustments, the influence of unpublished studies cannot be fully ruled out. Although the calculated CCA indicated only slight overlap, some individual reviews exhibited substantial overlap, suggesting that overlap may be unevenly distributed across the evidence base. Therefore, residual dependence among included studies cannot be entirely excluded.

Furthermore, the clinical heterogeneity inherent in combining neurodegenerative, vascular, psychiatric, and neurodevelopmental disorders under a single analysis is a conceptual limitation. While we performed disease‐specific subgroup analyses as the primary basis for conclusions, the overall pooled effect size should not be interpreted as a universal effect across all conditions. The substantial between‐disease differences argue against pooling across disorders for clinical decision‐making; thus, the overall estimate should be interpreted as exploratory and hypothesis‐generating. Future umbrella reviews should consider limiting to more homogeneous disease clusters (e.g., neurodegenerative disorders only) if sufficient evidence exists. Another limitation concerns the geographical distribution of the included meta‐analyses. The majority (22 of 29, 75.9%) were conducted in China, with the remainder from Europe, North America, and other Asia‐Pacific regions. This geographical concentration may affect the generalizability of our findings. Potential sources of bias include differences in clinical practice (e.g., concomitant medication use, rehabilitation standards), sociodemographic characteristics of patient populations, and potential publication or language bias favoring positive results from certain regions. Moreover, rTMS protocols and parameter selection may vary regionally without clear standardization. Future international collaborative efforts should prioritize multicenter trials across diverse populations to enhance external validity.

In summary, HF‐rTMS demonstrates consistent cognitive benefits in AD and VCI, but evidence for other disorders remains uncertain. While pooled analyses show favorable trends, low evidence certainty and heterogeneity limit reliability. Future research must prioritize large‐scale, pre‐registered RCTs with standardized parameter reporting and explore mechanism‐based, individualized strategies to facilitate clinical translation.

## 5 Conclusion

HF‐rTMS appears to improve cognitive function in Alzheimer's disease and vascular cognitive impairment, whereas its efficacy in other neuropsychiatric disorders remains uncertain due to limited evidence or null findings that require replication. Due to moderate pooled effects, low evidence certainty, and substantial heterogeneity, the strength of these conclusions is constrained. Future large‐scale, well‐designed randomized controlled trials with standardized protocols and individualized targeting are required to confirm long‐term benefits and guide clinical implementation.

## Author Contributions

WLL: conceptualization, investigation, formal analysis, Writing – original draft. HXC: methodology, validation, funding acquisition, supervision, Writing – review & editing. He also contributed to literature screening and data verification. Notably, as a cardiologist working within a psychiatric hospital's internal medicine department, his expertise in clinical epidemiology and biostatistics provided critical support for the meta‐analytic methods employed in this study. BJJ: investigation, data curation. HPY: software, visualization. All authors contributed to the conception and design of the study, approved the final version of the manuscript, and agree to be accountable for all aspects of the work.

## Funding

This work was supported by the Chengdu Medical Research Project (Grant No. 2021121; Principal Investigator: Xincheng Huang): “The Effect of Antipsychotic Drug Combination with High‐Frequency rTMS on cognitive impairment in First‐Episode Schizophrenia Patients and Its Psychopathological Mechanism.”

## Ethics Statement

This study is a systematic review and meta‐analysis of previously published data. As no new individual data were collected and no human participants were directly involved, ethical approval and informed consent were not required, in accordance with the Declaration of Helsinki.

## Conflicts of Interest

The authors declare no competing interests

## Supporting information




**Supporting Information**: brb371695‐sup‐0001‐Figures‐Tables.docx

## Data Availability

All data used in this umbrella review were extracted from published studies and are available within the article and its supplementary materials. The extracted dataset and statistical analysis files are available from the corresponding author upon reasonable request. If there are any reasonable requests, Xin‐cheng Huang can provide the summary analysis results (m13547836460@163.com).
