# Supplementary material for: Efficacy and Evidence Certainty of High‐Frequency rTMS for Cognitive Impairment Across Neuropsychiatric Disorders: An Umbrella Review
Source: Brain Behav. 2026 Aug 17;16(8):e71695. doi: 10.1002/brb3.71695 (PMC13482012; doi:10.1002/brb3.71695)
Supplement: Supplementary file 1 [file BRB3-16-e71695-s001.docx]

| **Supplementary Table 1. PRISMA 2020 Checklist** | | | |
| --- | --- | --- | --- |
| **Section and Topic** | **Item #** | **Checklist item** | **Location where item is reported** |
| **TITLE** | | |  |
| Title | 1 | Identify the report as a systematic review. | Page 1 |
| **ABSTRACT** | | |  |
| Abstract | 2 | See the PRISMA 2020 for Abstracts checklist. | Page 1. |
| **INTRODUCTION** | | |  |
| Rationale | 3 | Describe the rationale for the review in the context of existing knowledge. | Page 2 |
| Objectives | 4 | Provide an explicit statement of the objective(s) or question(s) the review addresses. | Page 2. |
| **METHODS** | | |  |
| Eligibility criteria | 5 | Specify the inclusion and exclusion criteria for the review and how studies were grouped for the syntheses. | Page 3-4 |
| Information sources | 6 | Specify all databases, registers, websites, organisations, reference lists and other sources searched or consulted to identify studies. Specify the date when each source was last searched or consulted. | Page 3 |
| Search strategy | 7 | Present the full search strategies for all databases, registers and websites, including any filters and limits used. | Page 3; Supplementary Table S2 |
| Selection process | 8 | Specify the methods used to decide whether a study met the inclusion criteria of the review, including how many reviewers screened each record and each report retrieved, whether they worked independently, and if applicable, details of automation tools used in the process. | Pages 3–4 |
| Data collection process | 9 | Specify the methods used to collect data from reports, including how many reviewers collected data from each report, whether they worked independently, any processes for obtaining or confirming data from study investigators, and if applicable, details of automation tools used in the process. | Pages 4 |
| Data items | 10a | List and define all outcomes for which data were sought. Specify whether all results that were compatible with each outcome domain in each study were sought (e.g. for all measures, time points, analyses), and if not, the methods used to decide which results to collect. | Pages 4 |
|  | 10b | List and define all other variables for which data were sought (e.g. participant and intervention characteristics, funding sources). Describe any assumptions made about any missing or unclear information. | Pages 4 |
| Study risk of bias assessment | 11 | Specify the methods used to assess risk of bias in the included studies, including details of the tool(s) used, how many reviewers assessed each study and whether they worked independently, and if applicable, details of automation tools used in the process. | Pages 4 |
| Effect measures | 12 | Specify for each outcome the effect measure(s) (e.g. risk ratio, mean difference) used in the synthesis or presentation of results. | Pages 4 |
| Synthesis methods | 13a | Describe the processes used to decide which studies were eligible for each synthesis (e.g. tabulating the study intervention characteristics and comparing against the planned groups for each synthesis (item #5)). | Pages 4 |
|  | 13b | Describe any methods required to prepare the data for presentation or synthesis, such as handling of missing summary statistics, or data conversions. | Pages 4 |
|  | 13c | Describe any methods used to tabulate or visually display results of individual studies and syntheses. | Pages 4-5 |
|  | 13d | Describe any methods used to synthesize results and provide a rationale for the choice(s). If meta-analysis was performed, describe the model(s), method(s) to identify the presence and extent of statistical heterogeneity, and software package(s) used. | Pages 4 |
|  | 13e | Describe any methods used to explore possible causes of heterogeneity among study results (e.g. subgroup analysis, meta-regression). | Pages 4-5 |
|  | 13f | Describe any sensitivity analyses conducted to assess robustness of the synthesized results. | Pages 4-5 |
| Reporting bias assessment | 14 | Describe any methods used to assess risk of bias due to missing results in a synthesis (arising from reporting biases). | Pages 4 |
| Certainty assessment | 15 | Describe any methods used to assess certainty (or confidence) in the body of evidence for an outcome. | Pages 4 |
| **RESULTS** | | |  |
| Study selection | 16a | Describe the results of the search and selection process, from the number of records identified in the search to the number of studies included in the review, ideally using a flow diagram. | Page 5; Figure 1 |
|  | 16b | Cite studies that might appear to meet the inclusion criteria, but which were excluded, and explain why they were excluded. | Figure 1; Supplementary Table S3 |
| Study characteristics | 17 | Cite each included study and present its characteristics. | Table 1; Pages 5–6 |
| Risk of bias in studies | 18 | Present assessments of risk of bias for each included study. | Figure 2; Page 6 |
| Results of individual studies | 19 | For all outcomes, present, for each study: (a) summary statistics for each group (where appropriate) and (b) an effect estimate and its precision (e.g. confidence/credible interval), ideally using structured tables or plots. | Figure 3; Figure 4; Pages 6–7 |
| Results of syntheses | 20a | For each synthesis, briefly summarise the characteristics and risk of bias among contributing studies. | Pages 6–7; Table 1 |
|  | 20b | Present results of all statistical syntheses conducted. If meta-analysis was done, present for each the summary estimate and its precision (e.g. confidence/credible interval) and measures of statistical heterogeneity. If comparing groups, describe the direction of the effect. | Pages 6–7; Figure 3 |
|  | 20c | Present results of all investigations of possible causes of heterogeneity among study results. | Pages 6–7; Figure 4 |
|  | 20d | Present results of all sensitivity analyses conducted to assess the robustness of the synthesized results. | Pages 6–7 |
| Reporting biases | 21 | Present assessments of risk of bias due to missing results (arising from reporting biases) for each synthesis assessed. | Table 2; Pages 6–7 |
| Certainty of evidence | 22 | Present assessments of certainty (or confidence) in the body of evidence for each outcome assessed. | Table 2; |
| **DISCUSSION** | | |  |
| Discussion | 23a | Provide a general interpretation of the results in the context of other evidence. | Pages 7–9 |
|  | 23b | Discuss any limitations of the evidence included in the review. | Pages 8–9 |
|  | 23c | Discuss any limitations of the review processes used. | Pages 8–9 |
|  | 23d | Discuss implications of the results for practice, policy, and future research. | Pages 9 |
| **OTHER INFORMATION** | | |  |
| Registration and protocol | 24a | Provide registration information for the review, including register name and registration number, or state that the review was not registered. | Page 3 |
|  | 24b | Indicate where the review protocol can be accessed, or state that a protocol was not prepared. | Page 3 |
|  | 24c | Describe and explain any amendments to information provided at registration or in the protocol. | Not applicable |
| Support | 25 | Describe sources of financial or non-financial support for the review, and the role of the funders or sponsors in the review. | Page 10 |
| Competing interests | 26 | Declare any competing interests of review authors. | Page 10 |
| Availability of data, code and other materials | 27 | Report which of the following are publicly available and where they can be found: template data collection forms; data extracted from included studies; data used for all analyses; analytic code; any other materials used in the review. | Page 10 |

*From:* Page MJ, McKenzie JE, Bossuyt PM, Boutron I, Hoffmann TC, Mulrow CD, et al. The PRISMA 2020 statement: an updated guideline for reporting systematic reviews. BMJ 2021;372:n71. doi: 10.1136/bmj.n71. This work is licensed under CC BY 4.0. To view a copy of this license, visit <https://creativecommons.org/licenses/by/4.0/>

**Supplementary Figure 1.** Supplementary Figure S1. Trim-and-fill funnel plot for publication bias analysis.

The open circles represent observed studies, while the gray squares represent imputed studies added to correct for asymmetry. The solid line indicates the adjusted overall effect size after trimming and filling.

**Supplementary Figure 2.** Forest plot after excluding the outlier study (Zhao_2024) in the VCI subgroup

**Supplementary Table 2. The complete search strategy for PubMed**

| **Search Set** | **Search Terms** |
| --- | --- |
| #1 | "Transcranial Magnetic Stimulation"[Mesh] OR Magnetic Stimulations, Transcranial OR Magnetic Stimulation, Transcranial OR Stimulations, Transcranial Magnetic OR Stimulation, Transcranial Magnetic OR Transcranial Magnetic Stimulations OR Transcranial Magnetic Stimulation, Paired Pulse OR Transcranial Magnetic Stimulation, Repetitive OR Transcranial Magnetic Stimulation, Single Pulse |
| #2 | "Schizophrenia"[Mesh] OR Schizophrenias OR Dementia Praecox OR Schizophrenic Disorders OR Disorder, Schizophrenic OR Disorders, Schizophrenic OR Schizophrenic Disorder |
| #3 | "Major Depressive Disorder"[Mesh] OR Depressive Disorders, Major OR Disorder, Major Depressive OR Disorders, Major Depressive OR Major Depressive Disorders OR Clinical Depression OR Depressions, Clinical OR Major Depression Disorder OR Depression Disorders, Major OR Disorders, Major Depression OR Depressive Disorder, Major OR Depression, Involutional OR Depressions, Involutional OR Melancholia, Involutional OR Melancholias, Involutional OR Psychosis, Involutional OR Psychoses, Involutional OR Paraphrenia, Involutional OR Paraphrenias, Involutional |
| #4 | "Bipolar Disorder"[Mesh] OR Bipolar Disorders OR Disorder, Bipolar OR Bipolar Mood Disorder OR Bipolar Mood Disorders OR Disorder, Bipolar Mood OR Mood Disorder, Bipolar OR Manic Depression OR Depression, Manic OR Depressions, Manic OR Affective Psychosis, Bipolar OR Bipolar Affective Psychosis OR Psychoses, Bipolar Affective OR Psychosis, Bipolar Affective OR Psychoses, Manic-Depressive OR Psychoses, Manic Depressive OR Manic-Depressive Psychosis OR Manic Depressive Psychosis OR Psychosis, Manic-Depressive OR Psychosis, Manic Depressive OR Depression, Bipolar OR Bipolar Depression OR Manic Disorder OR Disorder, Manic OR Manic Disorders OR Bipolar Disorder Type 1 OR Type 1 Bipolar Disorder OR Bipolar Disorder Type 2 OR Type 2 Bipolar Disorder |
| #5 | "Cognitive Dysfunction"[Mesh] OR Cognitive Dysfunctions OR Dysfunction, Cognitive OR Dysfunctions, Cognitive OR Cognitive Disorder OR Cognitive Disorders OR Disorder, Cognitive OR Disorders, Cognitive OR Cognitive Impairments OR Cognitive Impairment OR Impairment, Cognitive OR Impairments, Cognitive OR Mild Cognitive Impairment OR Cognitive Impairment, Mild OR Cognitive Impairments, Mild OR Impairment, Mild Cognitive OR Impairments, Mild Cognitive OR Mild Cognitive Impairments OR Cognitive Decline OR Cognitive Declines OR Decline, Cognitive OR Declines, Cognitive OR Mental Deterioration OR Deterioration, Mental OR Deteriorations, Mental OR Mental Deteriorations |
| #6 | "Alzheimer Disease"[Mesh] OR Alzheimer Syndrome OR Alzheimer-Type Dementia (ATD) OR Alzheimer Type Dementia (ATD) OR Dementia, Alzheimer-Type (ATD) OR Alzheimer's Diseases OR Alzheimer Diseases OR Alzheimers Diseases OR Alzheimer Dementia OR Alzheimer Dementias OR Dementia, Alzheimer OR Alzheimer's Disease OR Dementia, Senile OR Senile Dementia OR Dementia, Alzheimer Type OR Alzheimer Type Dementia OR Senile Dementia, Alzheimer Type OR Alzheimer Type Senile Dementia OR Primary Senile Degenerative Dementia OR Alzheimer Sclerosis OR Sclerosis, Alzheimer OR Dementia, Primary Senile Degenerative OR Dementia, Presenile OR Presenile Dementia OR Acute Confusional Senile Dementia OR Senile Dementia, Acute Confusional OR Alzheimer Disease, Early Onset OR Early Onset Alzheimer Disease OR Presenile Alzheimer Dementia OR Alzheimer Disease, Late Onset OR Late Onset Alzheimer Disease OR Alzheimer's Disease, Focal Onset OR Focal Onset Alzheimer's Disease OR Familial Alzheimer Disease (FAD) OR Alzheimer Disease, Familial (FAD) OR Familial Alzheimer Diseases (FAD) |
| #7 | Meta OR Systematic review |
| #8 | #1 AND (#2 OR #3 OR #4 OR #5 OR #6) AND #7 |

**Supplementary Table 3.** Overlap analysis of included meta-analyses

| **Review ID** | **Total Studies** | **Unique Studies** | **Overlapping Studies** | **Overlap Ratio** |
| --- | --- | --- | --- | --- |
| Chen_2024 | 19 | 13 | 6 | 31.58% |
| Chen_X_2023 | 41 | 31 | 10 | 24.39% |
| Chen_Y_2023 | 5 | 5 | 0 | 0.00% |
| Fu_2025 | 15 | 14 | 1 | 6.67% |
| Gong_2023 | 12 | 1 | 11 | 91.67% |
| He_2022 | 12 | 5 | 7 | 58.33% |
| Hou_2025 | 37 | 30 | 7 | 18.92% |
| Hsu_2015 | 14 | 12 | 2 | 14.29% |
| Jiang_Y_2019 | 32 | 21 | 11 | 34.38% |
| Lawrence_2017 | 14 | 12 | 2 | 14.29% |
| Le_2024 | 13 | 1 | 12 | 92.31% |
| Lu_2026 | 21 | 19 | 2 | 9.52% |
| Miller_2023 | 16 | 4 | 11 | 68.75% |
| Šimko_2022 | 16 | 2 | 14 | 87.50% |
| Teselink_2021 | 17 | 3 | 14 | 82.35% |
| Wang_2020 | 33 | 24 | 9 | 27.27% |
| Wang_2025 | 30 | 21 | 9 | 30.00% |
| Wang_M_2024 | 44 | 39 | 5 | 11.36% |
| Wang_T_2021 | 28 | 18 | 10 | 35.71% |
| Wang_X_2024 | 20 | 11 | 9 | 45.00% |
| Xiu_2024 | 17 | 9 | 8 | 47.06% |
| Xu_2022 | 10 | 7 | 3 | 30.00% |
| Zhang_2022 | 6 | 0 | 6 | 100.00% |
| Zhao_2024 | 13 | 2 | 11 | 84.62% |
| Zhao_2025 | 14 | 2 | 12 | 85.71% |
| Zhu_2024 | 10 | 0 | 10 | 100.00% |
| Total / CCA | 519 | 257 | 262 | CCA = 4.08% |

Note: Three meta-analyses (Jiang_L_2020, Jiang_Y_2020, Wang_2021) are not included because their primary study reference lists could not be retrieved (no DOI and no response from authors).
